# Peer mentoring in medical residency education: A systematic review

**DOI:** 10.36834/cmej.68751

**Published:** 2020-12-07

**Authors:** Helen Pethrick, Lorelli Nowell, Elizabeth Oddone Paolucci, Liza Lorenzetti, Michele Jacobsen, Tracey Clancy, Diane L. Lorenzetti

**Affiliations:** 1 Werklund School of Education, University of Calgary, Alberta, Canada; 2 Faculty of Nursing, University of Calgary, Alberta, Canada; 3 Department of Community Health Sciences, Cumming School of Medicine, University of Calgary, Alberta, Canada; 4 Faculty of Social Work, University of Calgary, Alberta, Canada; 5 Health Sciences Library, University of Calgary, Alberta, Canada

## Abstract

**Background:**

Medical residents may experience burnout during their training, and a lack of social support. This can impact their overall wellbeing and ability to master key professional competencies. We explored, in this study, the extent to which peer mentorship promotes psychosocial wellbeing and the development of professional competencies in medical residency education.

**Methods:**

We searched six databases (MEDLINE, EMBASE, PsycINFO, Academic Research Complete, ERIC, Education Research Complete) for studies on peer mentoring relationships in medical residency. We selected any study where authors reported on outcomes associated with peer mentoring relationships among medical residents. We applied no date, language, or study design limits to this review.

**Results:**

We included nine studies in this systematic review. We found that medical residents received essential psychosocial supports from peers, and motivation to develop academic and career competencies. Medical residents in peer-mentoring relationships also reported increased overall satisfaction with their residency training programs.

**Conclusions:**

Peer-mentoring relationships can enhance the development of key professional competencies and coping mechanisms in medical residency education. Further rigorous research is needed to examine the comparative benefits of informal and formal peer mentoring, and identify best practices with respect to effective design of peer-mentorship programs.

## Abstract (fr)

**Contexte:**

Les résidents en médecine peuvent ressentir un épuisement professionnel durant leur formation, et un manque de soutien social. Ceci peut affecter bien-être global et leur capacité à maîtriser des compétences professionnelles essentielles. Au cours de cette étude, nous avons examiné dans quelle mesure le mentorat par des pairs favorisait le bien-être psychosocial et l’acquisition de compétences professionnelles chez les médecins résidents.

**Méthodologie:**

Nous avons cherché dans six bases de données (MEDLINE, EMBASE, PsycINFO, Academic Research Complete, ERIC, Education Research Complete) des études sur le mentorat par des pairs pendant la résidence en médecine. Nous avons retenu toutes les études dont les auteurs avaient présenté des résultats associés aux relations de mentorat par des pairs chez les résidents en médecine, sans limite de date, la langue ou le devis.

**Résultats:**

Nous avons inclus 9 études dans cette revue systématique. Nous avons découvert que les médecins résidents recevaient un soutien psychosocial essentiel de la part des pairs et que ceux-ci les motivaient à acquérir des compétences universitaires et professionnelles. On a aussi constaté que les médecins résidents qui bénéficiaient d’un mentorat par des pairs étaient, de façon globale, plus satisfaits de leur programme de résidence.

**Conclusions:**

Le mentorat par des pairs peut favoriser l’acquisition de compétences professionnelles clés et de mécanismes d’adaptation au cours de la résidence en médecine. Il faudrait mener d’autres recherches rigoureuses pour comparer les avantages du mentorat informel à ceux du mentorat structuré et cerner les pratiques exemplaires de conception de programmes efficaces de mentorat par des pairs.

## Introduction

Mentorship is described as a developmental relationship between individuals with varying levels of experience.^1^ In a professional context, the transmission of tacit organizational knowledge and practical advice between mentors and mentees enables newcomers to develop career skills, form professional identities, and socialize into their work environments.^1-2^ Researchers have suggested that mentorship can provide individuals with a variety of relational and psychosocial supports including acceptance, collegiality, motivation, and opportunities to develop positive professional relationships.^3-5^

Many medical residency programs have incorporated competency-based educational curriculums into training programs that emphasize the development of core professional competencies.^6-7^ These include communication, professionalism, leadership, collaboration, and other competencies that can be difficult to adequately develop in classroom settings.^7-8^ Researchers have suggested that medical residents develop these skills through practice, and socialization into the medical profession, including interactions with peers; yet many residents may have limited opportunities to establish those supportive peer relationships that can advance these key professional competencies.^7-9^ While authors of prior reviews have examined the impact of senior mentors on medical students and early career professionals, few have explored the role of peers in promoting knowledge sharing, and the development of competencies or skills in medical residency education.^5,10-13^ Authors who have conducted research on peer mentorship in other contexts have argued that, through the provision of emotional and social supports, peers enable one another to adapt to new learning environments and develop as professionals.^14-15^

Medical residents can experience greater degrees of burnout and depressive symptoms than is typical of the general population.^16^ Burnout in medical residency can occur as a result of transitioning from medical student to resident roles, job stress, increasing workloads, a lack of workplace autonomy, inadequate financial remuneration, isolation from colleagues and peers, and an absence of support from supervisors.^9,17^ Researchers who have studied peer mentorship in graduate education and among healthcare professionals in workplace settings have found that peer mentoring increases socialization and reduces symptoms of burnout.^14,18-23^ In a randomized controlled study of the impact of a peer support group program for healthcare workers, Peterson et al. reported that “statistically significant intervention effects were found for general health, [and] perceived quantitative demands at work”.^22^ Other researchers have also noted that increased social or emotional support were associated with decreased burnout among college students and social workers.^19-20^

Medical residents’ attitudes towards residency education suggest that they value supportive learning cultures, and opportunities to develop ongoing friendships with peers.^24^ In a qualitative study of general practice residents in an extended residency program Agius and colleagues found that residents believed peer relationships were fundamental to that program’s success.^18^ Similarly, in a study of radiology residents’ career decision making, researchers reported that peers provided residents with information that informed decision making with respect to fellowship training.^21^

While many developmental benefits can result from engagement in peer mentorship, relatively little is known about the impact of peer mentoring in the context of medical residency education. In this systematic review, we explored the extent to which peer mentoring relationships support medical residents’ mental wellbeing, social connectedness, and the development of academic and career competencies or skills.

## Methods

We conducted a systematic review of the peer-reviewed literature on peer mentoring among medical residents. Our interdisciplinary research team comprised students and faculty members from education, library, medical education, nursing, and social work disciplines. This review was completed in November 2017 in accordance with the PRISMA and ENTREQ reporting guidelines.^25-26^ We previously published a protocol for this review in *BMC Systematic Reviews*.^27^ For the purposes of this review, we defined medical residents as any learners participating in post-graduate medical specialty training under the supervision of a senior physician.

We developed a comprehensive search strategy in consultation with a health sciences research librarian (DLL). We searched six medical and education databases: MEDLINE, EMBASE, PsycINFO, Academic Search Complete (EBSCO), Education Research Complete (EBSCO), and ERIC (EBSCO). Our searches combined terms relevant to three themes: 1) peers (e.g., peer, buddy, buddies), 2) mentorship (e.g., mentoring, mentors, mentees, protégés), and 3) medical residents (e.g., residents, house officers, registrars). We searched terms as both keywords (title/abstract) and subject headings as appropriate. We also scanned the reference lists of all included studies to identify additional relevant studies. We did not apply limits on date, language, or study design. The search strategy we implemented was reported in full in a previously published protocol for this study.^27^

### Inclusion/exclusion criteria

We included studies where authors reported on academic, career, or psychosocial outcomes associated with formal (assigned peer mentors) or informal peer mentoring relationships among medical residents. We excluded studies if: 1) the mentors were non-peers (i.e., faculty, staff, or professionals); 2) the peer mentors were not medical residents; 3) authors did not report outcomes; 4) it was not possible to separate medical resident outcomes from those of other study participants, or 5) it was not possible to isolate mentoring outcomes from those of other interventions. Teams of two reviewers (HP, LN, DLL) independently screened all study abstracts for inclusion, then independently assessed the full-text of included studies. We resolved any selection discrepancies through discussion or consultation with a third reviewer (EOP).

### Data extraction & quality assessment

One reviewer (HP) completed data extraction for the included studies, and two other authors (DLL, LN) verified the extracted data for consistency and accuracy. We extracted the following data from each study: basic study information (authors, study design, year of publication), study objectives, participant characteristics, outcomes associated with peer mentoring, and descriptive information on the design or implementation of formal peer mentoring programs. We used two quality appraisal tools (the Critical Appraisal Skills Program tool for qualitative studies, and the Joanna Briggs Institute (JBI) tool for cross-sectional study designs) to assess the quality of included studies.^28-29^ We modified the JBI tool to include an assessment of the extent to which authors addressed ethical considerations in their studies. We assessed mixed method studies with both tools. Teams of two authors (HP, LN, DLL) independently assessed the quality of eligible studies and resolved discrepancies through discussion. We did not exclude studies based on their quality.

### Data synthesis

Three authors (HP, LN, DLL) conducted a convergent thematic analysis of data extracted from included studies. Convergent thematic analysis is a process for identifying areas of convergence across qualitative, quantitative and mixed methods data.^30^ During this process, we applied qualitative thematic analysis techniques to transform and code study data, create concepts, identify areas of convergence across data, and combine concepts into higher-order themes.^30-31^ The authors then engaged in discussions to arrive at consensus on these themes. Due to the quality and heterogeneous nature of the included quantitative literature, we chose not complete a meta-analysis.

***Ethics:*** We did not require ethics approval to complete this study.

## Results

**Figure 1:**
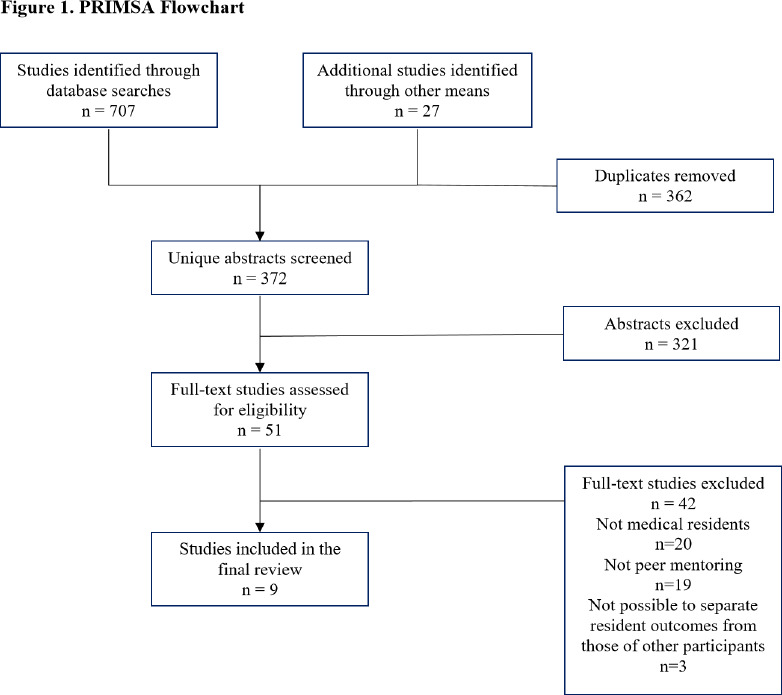
PRIMSA Flowchart

Our searches of electronic databases and reference list searching identified 372 unique studies (Figure 1). Of these, we included 9 studies (reported in 10 full-text articles) in this review (Table 1). The authors of included studies reported outcomes associated with formal peer mentoring programs (*n* = 7) or informal peer mentoring relationships (*n* = 2). The studies identified in this review comprised 7 cross-sectional studies (with no controls), 1 qualitative, and 1 mixed methods study (with no control). The quality of included studies ranged from medium to high (Table 1). While study quality was not a criteria for inclusion in this review, the most common quality issues we identified (as outlined in the quality appraisal tools previously noted) were: no explicitly mention of ethical concerns (*n* = 4); minimal description of study setting (*n* = 3); and failing to report on criteria for participant inclusion (*n* = 2) (Table 2, Table 3). In addition, we would add a uniform absence of control groups across studies.

**Table 1:** Descriptions of included studies

Study/ Country	Study Design	Study Objective	Participant Characteristics	Presence of Control	Program Elements	Outcomes
Chakravarti et al. 2017a; 2017b^37-38^ Canada	Quantitative	Evaluate the outcomes of the Anesthesiology Resident Wellness Program, including a peer mentoring component	28 anesthesiology residents (20 PGY1-3; 8 PGY4-5)	No Control	•One-to-One and Group Mentoring •Mandatory participation •Mentor matching (PG1-PG2 – minimum 1 year and PG1-PG5 for 2 weeks) •Work with/observe PG5 mentors in operating room for 2 weeks	•64% mentees ranked peer mentorship as the most valued component of this program
Eisen et al. 2013^32^ UK	Quantitative	Evaluate the benefits of peer mentoring for residents	62 pediatric residents (18 PGY1, 18 senior residents)	No Control	•One to One Mentoring •Voluntary participation •Mentor selection by mentees •Mentor training & ongoing support •Social events	•94% mentees reported that peer mentors provided a significant source of support. •100% of mentors improved their coaching and mentoring skills. •94% mentors and mentees improved their communication skills.
Hilliard et al. 2007^40^ Canada	Qualitative	Explore pediatric residents’ ethical conflicts and coping strategies	21 pediatric residents (5 PGY1, 8 PGY2, 3 PGY3, 5 PGY4)	No Control	N/A	•Mentees reported that peer mentors provided support in dealing with moral distress and ethical conflicts
Hoedebecke et al. 2014 ^35^ USA	Quantitative	Evaluate a peer-mentorship program to increase scholarly activity among residents	Family medicine residents (PGY1-3)	No Control	•One to One and Group Mentoring •Mentor matching •Peer manuscript review •Monthly group meetings to track progress over 1 year •Sharing information on publishing and conference submission opportunities	•Residents participation in scholarly activity (peer reviewed conference and manuscript submissions) increased from 16.7% (n=4) to 70.8% (n=17).
Obura et al. 2011^36^ Kenya	Mixed methods	Assess the impact of a group peer e-mentoring program among radiology residents.	10 radiology residents (3 PGY1, 2 PGY2, 2 PGY3)	No Control	•Group Mentoring (Facilitated) •Weekly meetings •Confidential Google Discussion board	•The program gave residents confidence to learn from colleagues (*M*= 4.2 on 5-point Likert scale) and helped them to feel more comfortable asking colleagues for advice (*M*=3.9 on 5-point Likert scale). •Qualitative feedback indicated that program participation enhanced residents’ knowledge sharing and sense of community
Parrott et al. 2006^39^ USA	Quantitative	Evaluate the impact of peer coaching in increasing residents’ use of five clinical microskills	12 residents (PGY2-3)	No Control	•One-to-One and Group Mentoring •Individualized peer coaching (2 weeks)	•Peer mentorship intervention group used significantly more total microskills per encounter than the control group (2.9 vs 1.9, *p* < 0.00029).
Prins et al. 2007^41^ Netherlands	Quantitative	Examine the association between residents' burnout and levels and types of psychosocial support	158 residents	No Control	NA	•Significant positive association (r=0.18, *p* < 0.05). between dissatisfaction with appreciative support from fellow residents and feelings of depersonalization
Vulliamy & Junaid 2013^33^ UK	Quantitative	Assess the feasibility and acceptability of a peer mentoring program among junior surgical residents	18 core surgical residents (9 PGY1, 9PGY2)	No Control	•One to One Mentoring •Voluntary participation •Mentor matching	•83% mentees received support with respect to preparation for examinations. •67% mentees received guidance on CV development and workplace assessments.
Webb et al. 2015^34^ UK	Quantitative	Assess the uptake and impact of a pilot peer mentoring program among residents	42 core medical residents (21 PGY1 21 PGY2)	No Control	•One to One Mentoring •Voluntary participation •Mentor matching •Mentor & mentee training •Mentorship agreements	•100% of mentors and mentees reported improved listening skills •86% of mentees reported improved communication skills •Most mentors and mentees believed that the program helped them to better manage work-related stress

Abbreviations: PGY: post-graduate year; indicates year of residency

**Table 2:** Quality Assessment of Quantitative Studies (JBI Cross Sectional Studies Checklist)

Study	Quality Score	Clear Inclusion Criteria	Detailed Setting Description	Valid / Reliable Exposure	Objective / Standard Measurement Criteria	Confounding Factor Identification	Valid / Reliable Outcome Measurement	Ethical Issue Consideration	Appropriate Statistical Analysis
Chakravarti et al. 2017a; 2017b^37-38^	7/8 Medium	Yes	Yes	Yes	Yes	No	Yes	Yes	Yes
Eisen et al. 2013^32^	7/8 Medium	Yes	Yes	Yes	Yes	No	Yes	Yes	Yes
Hoedebecke et al. 2014^35^	6/8 Medium	No	Yes	Yes	Yes	No	Yes	Yes	Yes
^*^Obura et al. 2011^36^	7/8 Medium	Yes	Yes	Yes	Yes	No	Yes	Yes	Yes
Parrott et al. 2006^39^	4/8 Low	No	No	Yes	Yes	No	Yes	Unclear	Yes
Prins et al. 2007^41^	5/8 Low	Yes	Unclear	Yes	Yes	No	Yes	Unclear	Yes
Vulliamy & Junaid 2013^33^	4/8 Low	Yes	Yes	Yes	Yes	No	Unclear	No	Unclear
Webb et al. 2015^34^	4/8 Low	Yes	Yes	Yes	Unclear	No	Unclear	Yes	Unclear

* mixed methods study; both CASP and JBI tool used to evaluate quality

**Table 3:** Quality Assessment of Qualitative Studies (Critical Skills Appraisal Programme Qualitative Checklist)

Study	Quality Score	Clear Aims	Appropriate Methodology	Appropriate Design	Appropriate Recruitment	Appropriate Data Collection	Researcher Participant Relationship Consideration	Ethical Issues Addressed	Rigorous Data Analysis	Clear Findings	Value of Research Stated
Hilliard et al. 2007^40^	10/10 High	Yes	Yes	Yes	Yes	Yes	Yes	Yes	Yes	Yes	Yes
^*^Obura et al. 2011^36^	9/10 High	Yes	Yes	Yes	Yes	Yes	No	Yes	Yes	Yes	Yes

* mixed methods study; both CASP and JBI tool used to evaluate quality

### Program implementations

We included seven studies describing the implementation and evaluation of formal peer mentorship programs for medical residents.^32-39^ The mentoring program models that were implemented in these studies included one-on-one mentoring,^32-34^ group-facilitated mentoring^36^ and interventions that included both approaches to peer mentorship.^35,37-39^ The objectives of these programs were to provide residents with opportunities to experience peer mentoring,^32-34^ or achieve specific goals including improving wellness,^37,38^ increasing scholarly activity,^35^ enhancing peer-teaching competencies,^39^ and improving problem solving skills.^36^ While peer mentors and mentees in one-on-one programs determined when and how often they would meet, those in group-facilitated programs usually met monthly with faculty facilitators (Table 1). Although many study authors did not include detailed descriptions of specific program activities in their reporting, four authors did comment on the inclusion of introductory social or training events as part of program structures, or ongoing social, learning, and team-building events (Table 1).

### Outcomes

Studies exploring the impact of formal peer-mentoring programs on competency-building and psychosocial wellbeing found that peer mentoring had a positive effect on a variety of psychosocial and career outcomes. Eisen et al. reported that 94% of paediatric residents who participated in a peer-mentoring program in the UK improved their communication skills.^32^ In a survey of peer mentoring among core medical residents, Webb et al. found that 100% of residents who participated in peer mentoring relationships reported improvements in listening skills and increased understanding of academic and personal challenges faced by their mentors or mentees.^34^ Authors of two studies exploring the impact of peer-mentoring programs on academic and career outcomes reported that participation in these programs enabled residents to achieve a number academic and career milestones, including improvements in exam results, scholarship, success in grants competitions, and an increased ability to secure promotions.^33,35^ Finally, four studies found that peer-mentoring programs contributed to an increased sense of community among residents, resulting in the creation of cohesive and less competitive peer groups.^32,37,36,^ ^38^

While no study authors specifically reported on burnout, three studies did find that peer mentors promoted mental wellbeing among medical residents.^34,37,38,40^ Webb et al. reported that both mentors and mentees believed that peer relationships helped them to manage work related stress.^34^ Hilliard et al. found that peer mentors better enabled residents to manage work-based ethical conflicts and moral distress.^40^ Finally, in an evaluation of a multi-component program to improve mental wellbeing of anesthesiology, Chakravarti et al. found that 64% of residents valued opportunities to engage in conversations with peers on substance abuse and addictions within the medical profession, and 50% reported that they benefited from discussions that focused on transitioning into new professional roles.^37-38^ Residents in this program also indicated they developed an appreciation for strategies to improve their psychosocial wellbeing through self-care, work/life balance, and developing strong peer teams.^37-38^

## Discussion

We undertook this systematic review to explore the extent to which peer mentoring relationships relate to psychosocial wellbeing and the development of professional competencies among medical residents. Our findings suggest peer mentoring can further the development of medical residents’ communication skills, promote academic and scholarly success, and support ongoing career development. We also found evidence to suggest that peer mentorship promotes the development of a sense of community among residents and may enhance coping skills and psychosocial wellbeing. However, as the quality of the studies identified in this review was variable and none included a control group, we would emphasize the need for a degree of caution in the interpretation of these findings.

Mentoring within the medical professions can facilitate career progress, academic guidance, and increased research productivity.^5,14^ While our review aligns with previous research reporting on the benefits of mentorship in medical education, it is unique in its focus on the role of peer mentors in developing interpersonal and coping skills, and academic and career competencies among medical residents.^12,15,23,42^

A culture of supportiveness amongst peers in medical residency may help to address the psychosocial needs of medical residents as they transition to new roles, and increasingly challenging learning environments.^11^ Recent developments in medical education have emphasized the importance of medical residents cultivating soft skills throughout their training.^13^ These include communication, collaboration, and leadership competencies, all of which may not be readily mastered in classroom settings. The findings from this review suggest that peer mentoring relationships can also encourage and enable medical residents to develop and enhance their capacity in these and other essential skills.^13^

Our review has strengths and limitations. We conducted a comprehensive and rigorous review of the peer-reviewed literature, including quantitative, qualitative, and mixed methods data in our analysis. However, a search of the grey literature may have identified additional studies of relevance to this review. Further, while our review suggests that peer mentoring is an essential component of medical residency education, variability in study quality, and the absence of controlled, longitudinal studies may limit the strength and generalizability of the conclusions that may be drawn from our findings. Finally, we found no empirical evidence of the long-term effects of these relationships, or the extent to which specific program designs may promote or hinder residents’ professional development or psychosocial wellbeing.

Further research is needed to address important gaps in the current literature and inform the design of formal peer mentorship programs that can be of benefit to medical residents. Rigorous controlled studies of the effects of peer mentoring are required to reduce the risk of study bias and confirm and contextualize existing evidence on the impact of peer mentorship in medical residency education. Further, both qualitative and longitudinal study designs of peer mentorship experiences are needed to identify the peer-mentoring preferences of medical residents. These data could be leveraged to support the design and implementation of formal peer mentorship programs that focus on the development of key professional competencies in medical residency education, and the psychosocial wellbeing of medical residents.

## Conclusions

While further investigations regarding the effectiveness of specific peer mentoring program designs and in-depth explorations of the impact of peer mentoring relationships among medical residents are warranted, this review found that peer-mentoring relationships may enhance the development of key professional competencies and coping mechanisms in medical residency education.
